# Skip the Alignment: Degenerate, Multiplex Primer and Probe Design Using K-mer Matching Instead of Alignments

**DOI:** 10.1371/journal.pone.0034560

**Published:** 2012-04-02

**Authors:** David A. Hysom, Pejman Naraghi-Arani, Maher Elsheikh, A. Celena Carrillo, Peter L. Williams, Shea N. Gardner

**Affiliations:** 1 Computations, Lawrence Livermore National Laboratory, Livermore, California, United States of America; 2 Physics and Life Sciences, Lawrence Livermore National Laboratory, Livermore, California, United States of America; 3 Joint Genome Institute, Lawrence Berkeley National Laboratory, Walnut Creek, California, United States of America; Naval Research Laboratory, United States of America

## Abstract

PriMux is a new software package for selecting multiplex compatible, degenerate primers and probes to detect diverse targets such as viruses. It requires no multiple sequence alignment, instead applying k-mer algorithms, hence it scales well for large target sets and saves user effort from curating sequences into alignable groups. PriMux has the capability to predict degenerate primers as well as probes suitable for TaqMan or other primer/probe triplet assay formats, or simply probes for microarray or other single-oligo assay formats. PriMux employs suffix array methods for efficient calculations on oligos 10-∼100 nt in length. TaqMan® primers and probes for each segment of Rift Valley fever virus were designed using PriMux, and lab testing comparing signatures designed using PriMux versus those designed using traditional methods demonstrated equivalent or better sensitivity for the PriMux-designed signatures compared to traditional signatures. In addition, we used PriMux to design TaqMan® primers and probes for unalignable or poorly alignable groups of targets: that is, all segments of Rift Valley fever virus analyzed as a single target set of 198 sequences, or all 2863 Dengue virus genomes for all four serotypes available at the time of our analysis. The PriMux software is available as open source from http://sourceforge.net/projects/PriMux.

## Introduction

Viral species detection can be difficult when high levels of strain variation have evolved. Substantial investment is required to obtain adequate sequence data to represent the known diversity and to design conserved, species specific signatures [1], [2], [3]. Many published PCR-based signatures are not robust, and in a computational analysis of dozens of published signatures, over 60% of the viral signatures analyzed failed to detect all desired targets based on available sequences [4]. While methods like sequencing and microarrays can overcome this problem and detect a wide range of viruses of diverse strains and unanticipated species [5], PCR-based assays aimed at detecting one or several species are faster, less expensive, and more sensitive if detection is limited to a small number of possible organisms. As the amount of sequence data skyrockets with advances in sequencing technology, signature design software must scale up to keep pace.

Designing primers for sets of diverse target sequences typically starts with Multiple Sequence Alignment (MSA) to identify the most conserved regions for primer design [6], [7], [8], [9]. Usually, a clear block of conserved sequence is needed, requiring high quality alignments of fairly similar sequences. Obtaining a good MSA can be a bottleneck in terms of compute time and memory, confounded by sequence rearrangements, distinct subgroups, outliers, and mixtures of sequences in both the forward and reverse complement directions (e.g. in Genbank, Hanta virus Andes Maporal HV-97021050 segments M and S are in the reverse complement direction from other sequences in the genus, and should be changed to the reverse complement direction prior to an alignment). Algorithms typically run a sliding window over an alignment to identify primer-length regions with the fewest sequence variations, and a single window that minimizes degeneracy over the targets is identified. If the target sequences fall into distinct (even non-homologous) subgroups with different conserved regions, that is, no single window is the best for all subgroups, then the degeneracy of any primer selected to minimize degeneracy across all subgroups may be much higher than that of different degenerate primer windows from the most conserved locations within each subgroup. But identifying homologous subgroups and identifying outlying sequences (or sequences provided in the reverse complement direction) requires some analysis to curate sequences prior to primer prediction. This may not be an option in high throughput, automated primer prediction settings for which manual, expert curation is simply not feasible. “Eye strain” and “error prone” are terms we associate with manually examining alignments of hundreds to thousands of sequences, each thousands of bases in length.

Consequently, we decided to avoid sequence alignment altogether, instead applying k-mer frequency patterns to guide primer selection, where a k-mer is a string, or oligo, of length k. In previous work [1], we described the Multiplex Primer Prediction software (MPP), that prototyped a k-mer primer selection approach. Target sequences could have no or very limited homology, and the algorithm found combinations of primers to detect all targets, applying heuristics to minimize the number of primers required. MPP computed primers and/or probes by operating on sets of k-mers (oligos of length k) shared by multiple sequences in either the forward or reverse complement direction, and hence bypassed the MSA phase. However, MPP required that the oligos be exact matches, i.e, did not contain degenerate (mismatching) bases. We found that it could find near-minimal primer sets to cover hundreds of divergent viral genomes (for example, all sequences in a family) but in many cases there were still too many primers required to amplify all target sequences to be feasible at the bench. There was an obvious need to consider degenerate bases, and to do so without resorting to an MSA.

Therefore, we built PriMux employing the following approach. After initial k-mer enumeration from all target sequences using storage and memory-efficient suffix array methods (McIlroy, T.M. and McIlrow, M.D., Sarray, a collection of Suffix-array functions. http://www.cs.dartmouth.edu/~doug/sarray/ Copyright (C) Lucent Technologies) – k-mers are ranked by conservation among the targets. The most conserved k-mers are filtered for optimal primer or probe characteristics such as Tm, GC%, avoidance of homopolymer runs, homodimers, hairpin folds, and repetitive sequence. Conservation is then re-calculated with vmatch (http://www.vmatch.de), allowing up to a user-specified number of mismatches, allowing multiple similar k-mers to be condensed into a single k-mer with degenerate bases which detects all of the sequences detected by any of the variants. These degenerate k-mers are then re-ranked by conservation, i.e. largest number of targets detected. All k-mer to target sequence comparisons are performed in both the forward and reverse complement directions, so that target orientation does not confound the algorithm.

Several alternative greedy algorithms are then employed to select alternative sets of k-mer forward/reverse primer pairs. A “min” algorithm picks the most conserved primer pairs that will detect the *outlying, least conserved targets* first. A “max” algorithm picks the most conserved primer pairs that will detect the *most conserved targets first*. A “combo” algorithm does a combination of “min” and “max”. The algorithm that yields the fewest primer pairs that detect all targets can differ for various target sets, but “max” almost always performs best. The other advantage of the “max” algorithm is that if only a majority rather than all targets must be detected, one can use primers from the top of the list, and drop off those at the bottom which detect the minority, outlying sequences.

In addition, to achieve multiplex compatible primer sets, if nearest neighbor hybridization free energy calculations predict that a primer might dimerize with other primers already selected, it is dropped and the next most conserved primer is considered. Because there may be alternative, equivalent primers that can be selected at each step, different primer sets can be designed, and the user can tell the software how many sets are desired. This is advantageous since, if one set fails in lab testing, there are back-ups. The alternative sets may differ in size since the algorithms follow a greedy heuristic and do not compute a true optimum, as the minimal set degenerate primer selection problem has been proven to be NP complete [7].

**Figure 1:**
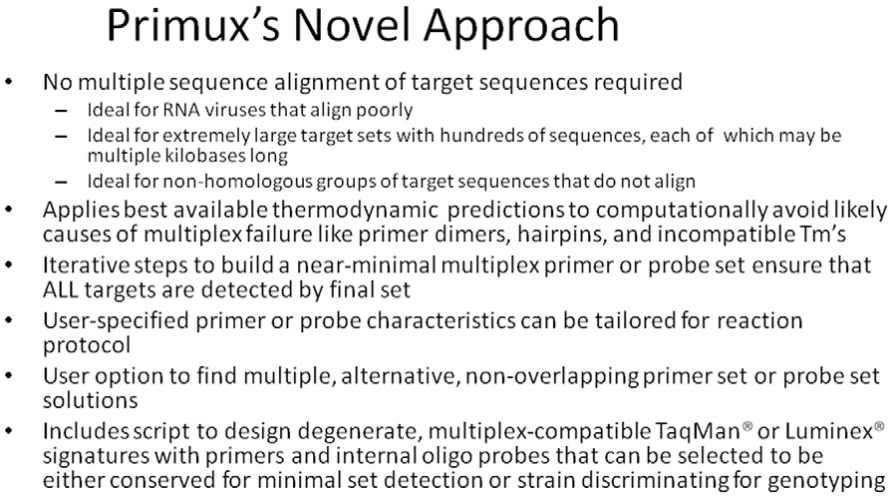
Text box listing important aspects of PriMux.

From the amplicons generated by a primer set, PriMux can design conserved probes to detect all targets with the fewest probes, or genotyping probes to maximize the target discrimination. The resulting primer and probe “triplets” are suitable for TaqMan®, Luminex/BioPlex®, or other similar assays, or probes can be designed for platforms like microarrays in which primers are not necessary. The probe selection process starts by computing all the k-mers on the amplicons and ranking by conservation, filtering by Tm, GC%, etc. as for the primers, and recalculating conservation allowing mismatches. For the fewest probes to detect all targets, the most conserved probes are selected for each target. For genotyping probes, the least conserved probes are selected for each target. PriMux is the only software to our knowledge that designs sets of conserved degenerate primers *and/or probes*. Many of the key features of PriMux that distinguish it from other primer prediction softwares are listed in Figure 1.

To demonstrate the software, we predict primer+probe triplets for 3 example target sets: Rift Valley Fever virus (RVFV) run on each segment separately; RVFV run on all segments together; and 2863 Dengue virus genomes. Primer/probe triplets with conserved probes were predicted for each (L, M, S) segment of RVFV and tested in the lab for sensitivity and specificity against target, background, and near neighbor viruses. 54, 60, and 84 sequences were available for the L, M, and S segments, respectively. To demonstrate that curation of input sequences into separate homologous groups for each segment is not necessary prior to running PriMux, we predicted a multiplex set of primers and probes such that all 198 L, M, and S sequences would be detected by at least one triplet in the multiplex. In addition to the ease of preparing the sequence input file (e.g. one can download the sequences under a taxonomy node without curation into L/M/S elements), another advantage of running the segments as a single input means that the solution set specifically avoids any primer dimer interactions predicted by nearest neighbor free energy calculations that could be possible if primers are combined in a single reaction from separate runs of PriMux on each segment. Finally, we designed a set of multiplex triplets for all sequenced full-length genomes of all serotypes of Dengue virus to show that PriMux can predict a reasonably small, multiplexed set of primers and probes for a large target set (2863 sequences) with very low homology and poor alignment.

## Methods

**Figure 2:**
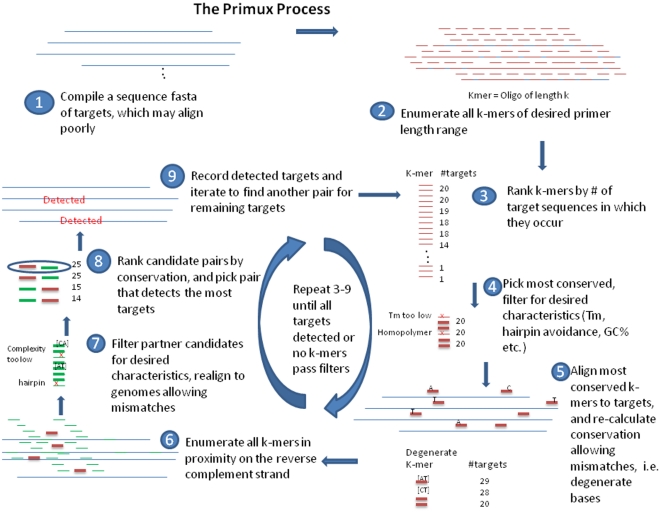
Diagram of the PriMux approach.

Figure 2 charts the approach employed by PriMux. The PriMux pipeline is implemented as a series of modules (i.e., scripts and executables) whose inputs and outputs are tied together via the file system. Compute intensive modules are coded in C ++. Other modules (primarily those involved in parsing and invoking third-party executable) are coded in python or perl. The PriMux pipeline for computing forward/reverse primer pairs is run by invoking a python script, **runme2.py**, which takes a single command line parameter, the name of an options file.

The options file specifies all settings relevant to an experiment, and a number of commonly modified options are shown in Table 1. These include the name of the input fasta-formatted file; settings to be passed to UnaFold [10]; the number of permitted degenerate base pairs in the primers; etc. Options files follow a simple text-based format, e.g;

#this is a comment

# directory in which results are stored

-dir = results

#min, max and increment for computing kmers

-min_kmer_len = 18

-max_kmer_len = 22

-kmer_len_inc = 1

**Table 1:** A subset of the most commonly changed user-specifiable parameters in the options file.

Selected User Parameters	Meaning
maxPolyX	maximum number of homopolymer bases allowed in a primer or probe
primer_selection_iterations	number of alternative, non-overlapping primer sets to attempt to find
probe_selection_iterations	number of alternative, non-overlapping probes to attempt to find
max_mm	maximum allowable number of degenerate bases per primer or probe
min_kmer_len	minimum primer or probe length allowed
max_kmer_len	maximum primer or probe length allowed
min_amplicon_length	minimum amplicon length allowed
max_amplicon_length	maximum amplicon length allowed
min_hairpin_dG	minimum hairpin free energy allowed
min_primer_dimer_dG	minimum free energy of primer dimers (including homodimers)
min_tm	minimum Tm allowed of primer or probe
max_tm	maximum Tm allowed of primer or probe
min_percent_gc	minimum % GC allowed
max_percent_gc	maximum % GC allowed
min_dist_mm_to_3prime_end	no degenerate bases are allowed closer than this to the 3′ end of a primer or probe

### Third party codes

In addition to in-house code, PriMux uses the third party codes: ssarray.tar (http://www.cs.dartmouth.edu/~doug/sarray/), UNAFold [10], Vmatch (Stephan Kurtz: The Vmatch large scale sequence analysis software, http://www.vmatch.de).

### Terminology

A **k-mer** is a string, or sub-sequence, of length k. Given a set of DNA sequences, **TopN** refers to the set of the *N* most frequently occurring k-mers. Although a k-mer may occur multiple times in a single DNA sequence, for the purposes of this work we tabulate TopN on the basis: a k-mer appears at least once in a DNA sequence (i.e., multiple occurrences are not significant). More expansively, a TopN set is computed as follows. Given a set of genomes and a value *N*, we compute the most frequently occurring kmers across all genomes.

**BottomN** is analogous to TopN, but refers to the set of least frequently occurring k-mers. TopN sets are used when computing a minimal set of PCR signature and probes, while BottomN sets are used when computing genotyping probes.

A **SuperSet** is a data structure that contains a k-mer, along with a listing of where the k-mer occurs in a given set of DNA sequences. A SuperSet also lists the locations of the degeneracies (if any) for each occurrence. Additionally, a SuperSet contains a field indicating if its k-mer is to be used in the forward, reverse complement, or either direction (f/r/e). To make this more concrete, an example SuperSet contains the following information:

#the kmer, with no degeneracies

ACTGTCTAGCA

#the kmer occurs in sequence 3, with the degenerate in position one filled by ‘C’

**C**CTGTCTAGCA :: 3 :: 1, C;

#the kmer occurs in sequence 5, with degenerate in position 1 filled by ‘T’ and degenerate position 6 filled by ‘A’:

**T**CTGT**A**TAGCA :: 5 :: 1, T ; 6, A

#the kmer occurs in sequence 6 with no degeneracies

ACTGTCTAGCA :: 6

A **PrimerPair** is a data structure that contains two SuperSets such that the k-mers in the SuperSets represent a PCR signature. This means that the distance between the two oligos is between the minimum and maximum amplicon length; and that both oligos have passed all filters (dimerization, melting temperature, etc; see below).

### Module and Pipeline Descriptions

**Figure 3:**
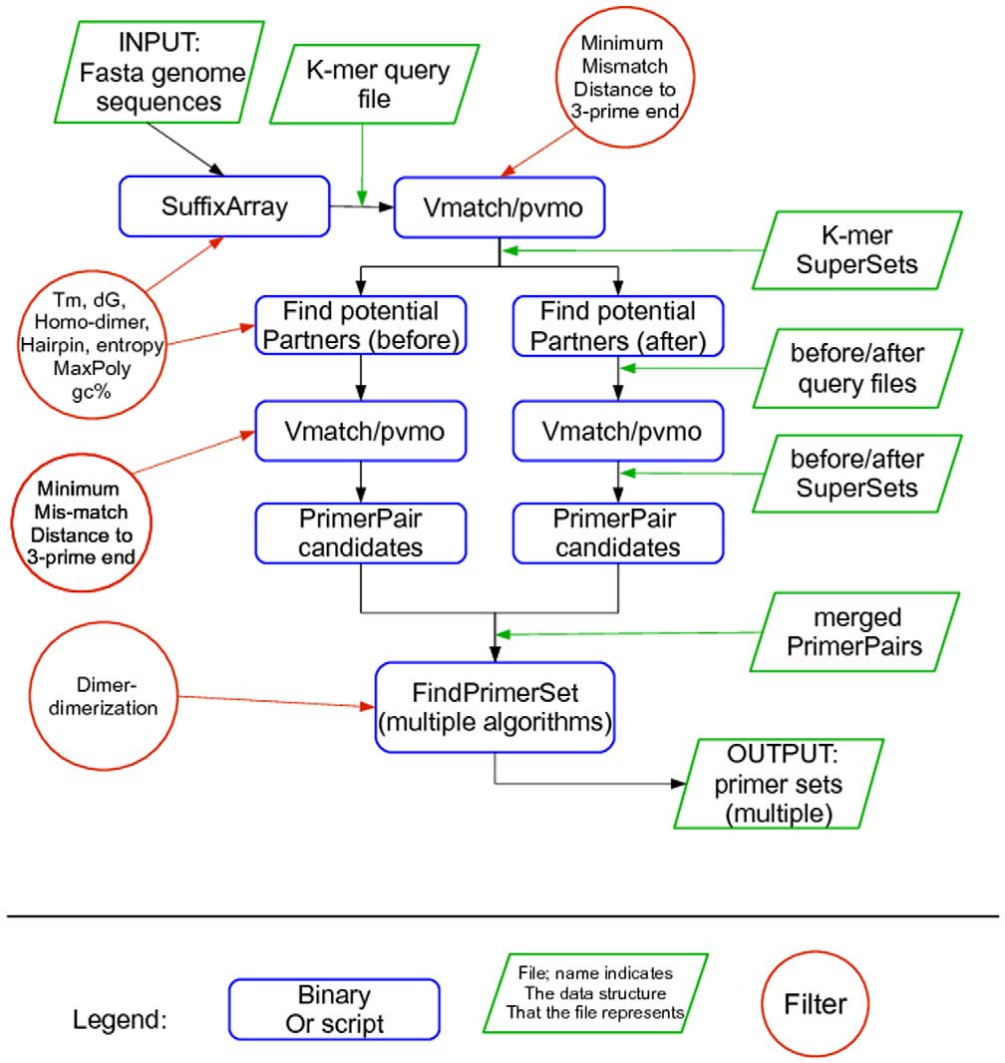
Diagram of the PriMux software workflow.

Figure 3 contains a flow diagram for PriMux's primer finding pipeline. The probe finding pipelines use many of the same modules.

The **SuffixArray** module computes the TopN and/or BottomN k-mers for a set of genomes and a given *k* (or range of *k*). This module uses the C-based Suffix-array code sarray as its compute-intensive kernel. The output set of kmers, which are written to a fasta-formatted file, are non-degenerate. We call the output the *k-mer query file*. In the options file, a user may specify a “topN_min” minimum number of k-mers representing each target for the query file. This ensures that each sequence is covered by at least topN_min k-mers. PriMux runs faster and uses less memory with smaller values of topN_min, because it considers fewer k-mers and k-mer combinations. The **Vmatch** module uses S. Kurtz's executables as its computational kernel. It takes as input the k-mer query file and an integer that specifies the maximum permitted number of degenerate base pairs, and outputs a file formatted as described at http://www.vmatch.de.

The **pvmo** module parses the output from Vmatch and constructs and writes to file a set of SuperSets. We refer to this as the *k-mer SuperSet file*.

The **findPotentialPartners** module takes as input the SuperSet file from the previous module. For each position of every k-mer in every SuperSet file it computes a set of ***potential partner*** k-mers. A potential partner is a k-mer that is within the required distance (between the desired minimum and maximum amplicon lengths, as specified in the options file) such that the k-mer and its potential partner form a pair of primers. The potential partners are generated using the **SuffixArray** module, and query files are written as described above. We search in both before and after directions to find potential partners. Hence we end up with two query files, the *before query file* and *after query file*. As above, the before and after query files are processed using **Vmatch**, and parsed to form sets of *before* and *after SuperSets*.

The **computePrimerPairCandidates** module takes as input the *k-mer SuperSet file* and the *before* and *after SuperSet files* and outputs a set of PrimerPairs. Instead of outputting all possible PrimerPairs, we down-select to ensure that we output a relatively small number (perhaps thousands), that the selected PrimerPairs cover the largest possible number of genomes, but that every genome is covered by a minimal number of PrimerPairs.

The **findPrimerSet** modules take as input a set of PrimerPairs and output one or more (ideally minimal) set(s) of primers suitable for PCR-based amplification of the target genomes. Each PrimerPair in the input set has already been determined to be a valid PCR signature. The goal of these modules, then, is to select a set of PrimerPairs such that (i) every sequence in the target set can be amplified by at least one forward/reverse complement pair of primers; (ii) the resulting set of primers is minimal.

As noted above, we have devised several heuristic algorithms that accomplish this aim. Our ***min*** approach picks a set of k-mer forward/reverse pairs based on the criterion: select primers that will detect the *least* conserved targets first. Our ***max*** algorithm picks a set of k-mer forward/reverse pairs based on the criterion: select primers that will detect the *most* conserved targets first. Our ***combo*** algorithm utilizes both min and max strategies. First, one primer pair is selected that covers the most conserved targets; this is the max phase. Then, additional pairs are added using the min strategy, until all targets are covered by at least one primer pair.

Each algorithm also takes as input a number of requested iterations. For each iteration, the algorithms work as described above, but exclude from consideration any primers that overlap with PrimerPairs that were previously selected.

After a set of primers has been found to detect all targets, it is fixed to remove any extra primers that result in redundant coverage of any targets, which can occur due to the greedy algorithm used. The final sets of primers are reported in files beginning in the word “fixed”. Redundant coverage can occur, for example, if the 1st selected primer-pair covers sequences 1–4; then 2nd covers 5, but also covers 1 and 2; the 3rd covers 6, but also covers 3 and 4. So now the 1st primer is not needed.

The primer prediction modules are called by the wrapper script runme2.py which takes an options file as input. The target fasta input is indicated in the options file, as are the desired primer parameter specifications. A Quickstart guide and html documentation are included in the software distribution.

#### Probes

The **findConservedProbes.py** script finds near-minimal sets of probes to match all targets. It is identical to the findPrimers.py pipeline through computation of the k-mer SuperSet file. It then applies a max algorithm that computes one or more minimal sets of k-mers (the probe set), such that each sequence in the input file contains a probe from the set. This script computes degenerate probes.

The **findGenotypeProbes.py** script attempts to find sets of strain discriminating probes. That is, given a set of target sequences, it attempts to generate a set of probes to discriminate the targets to the maximum level allowed by the sequence diversity. This pipeline is similar to findConservedProbes.py, however, instead of working with the TopN k-mers it works with the BottomN sets. This script does not allow degenerate probes, since the goal is maximum discrimination.

### Filters

At various stages in the pipeline, filters are applied to eliminate k-mers that violate constraints specified in the options file. Filters include:

- **UNAFold**: does a k-mer adhere to the specified minimum and maximum melting temperatures, hairpin, and homdimerization constraints?
- **GC percent**: does a k-mer's GC percent fall within the specified minimum and maximum constraint?
- **entropy**: a k-mer's entropy must be above the specified minimum. Entropy is computed as 
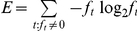
 where f_t_ = f_AAA_, …, f_TTT_ are the frequencies of each of the 64 possible trimers in the k-mer sequence, calculated as the number of occurrences of each trimer divided by the total number of trimers in the sequence. The sum is over the trimers t with f_t_>0. The entropy filter eliminates repetitive, low complexity sequence that tends to function poorly as primers.
- **min_dist_mm_to_3prime_end**: a k-mer position in a SuperSet is deleted if it contains a degenerate base pair that is within min_dist_to_3prime-end base pairs of the 3prime end.
- **maxPolyX**: the maximum permitted number of identical sequential base pairs in a k-mer. By example, if maxPolyX = 3, the kmer ACTTTT would be rejected due to the sub sequence ‘TTTT.’
- **dimerization:** when selecting the primer sets (per findPrimerSet above), does each potential primer fall within the (UNAFold calculated) dimerization constraints for all previously selected primers?

#### Other Features and Output

Users may specify the number of alternative primer or probe set solutions with the variable in the options file indicating the number of primer or probe selection iterations. Primers are selected so as not to overlap with those already chosen in a previous iteration of the same algorithm (max, min, or combo), to avoid nearly identical solutions that differ by only a few bases at the end of an oligo. However, if no more candidate k-mers pass the filters, then the program ends and fewer than the desired number of solutions may be found, or some of the target sequences may not have primer pairs that detect them. The likelihood that this occurs depends on the stringency of the filters, the number of solutions already chosen, and the size of the target set (due to dimerization avoidance). Relaxing the filters (e.g. enlarging Tm or length ranges, allowing longer homopolymers, decreasing the minimum allowable dimerization free energy, etc.) or increasing topN_min and re-running may increase the number of targets detected or the number of solutions found.

The file names that end with “amplicons” list the amplicons and positions generated by every primer pair for every target, for the solution indicated in the file name. For example, “fixed_primers_mm_max_rep = 0_amplicons” is the first solution with the max algorithm. Targets for which primer pairs cannot be found are indicated with “NO AMPLICONS FOUND”. The file names that end with “amplicon_distribution” list the size distributions of amplicons as could be observed in an electrophoretic banding pattern. When running findConservedProbes.py or findGenotypeProbes.py, targets detected by each probe are listed in the target_coverage.[solution#] files in the probe results directory.

#### Designing Primer/Probe Triplets for TaqMan® or Luminex® assays

A wrapper program called run_PriMux_triplet is included to automatically find multiplex “triplet” signatures, each with three components, 2 primers and a probe, to insure coverage of all target sequences. This script calls the runme2.py script to design sets of conserved, multiplex compatible degenerate primers. Then it calls another perl script to extract the predicted amplicon sequences, removes the primer sequences from the amplicon ends, and calls findConservedProbes.py to predict a set of conserved probes such that all targets are amplified by one or more primer pair+probe combination. Optionally, it also can run findGenotypeProbes.py. It takes separate options files as input for the primers and probes, since the Tm, length, and other specifications will be different. It also prints a “*sorted” file with a ranked list of the number of targets amplified by every primer pair combination predicted to yield a product, so a user can quickly see patterns of highly conserved primer pairings vs pairs that only pick up a few outlying sequences. The script also labels primers as forward or reverse based on the majority vote: in the multiplex, a primer could act as both a forward and a reverse primer in different reactions. This ranked list gives every primer sequence variant without degenerate bases, so that a user can see variant frequency information at the degenerate positions. This could be useful if a user desires to reduce primer numbers or degeneracy by omitting primers that only detect rare variants.

### Computational Examples

Signatures for the L, M, and S segments of RVFV were designed from all available complete segment sequences using run_PriMux_triplet. The sequence identifiers and parameter options used as input to PriMux are available upon request. We required primers to be 18–22 nt long, Tm = 60–65°C, up to 3 degenerate bases no closer than 3 nt from the 3′ end, no more than 4 bases in a homopolymer repeat, GC% of 20–80%, and produce amplicons 80–250 bp. PriMux probes could have up to 3 degenerate bases that could be anywhere on the probe, length 18–30 nt long, Tm = 68–73°C, no more than 4 bases in a homopolymer repeat, and GC% of 20–80%.Thus, we found conserved primer and probe combinations for each segment to detect all sequences of that segment. Sequences from one of the several possible solutions for each segment are listed in Table 2, and these were tested in the lab as described below, and compared with signatures that we had previously designed and tested using the standard approach of finding consensus regions from a multiple sequence alignment as described in [11]. Briefly, the standard (non-PriMux) signature design method entailed comparing all available complete genomes of different strains of RVFV using MSA. All available complete or partial RVFV genomes were computationally examined to identify sequence regions that were conserved among all sequenced isolates of RVFV but also able to uniquely identify RVFV when compared against all available genomic sequence data. A final *in-silico* screening was performed to verify that signatures were predicted to detect the RVFV strains and not to detect any non-pathogen targets. The conserved/unique sequence information was used to develop candidate signatures that met Real-Time RT-PCR assay chemistry requirements. No degenerate bases were used, since the sequence availability at the time those signatures were designed was not sufficiently diverse to require the use of degenerate bases in primers or probes.

**Table 2:** Rift Valley Fever (RVF) Degenerate Assay Signatures.

Sig-natureName	Forward PrimerSequence	Reverse PrimerSequence	Probe Name	Conserved Probe Sequence
RVF_S	CTTGGCATCCTTCTCCCAG	CAAGCAGTGGACCGCAAT	RVF_S_C1	TTGARCAGTGGGTCCGAGAGTTTG
			RVF_S_C2	TGCTTATCARGGRTTTGATGCCCGTAG
RVF_M1	AGCCATCATTGCTGCYGATG	AGGCTGGAAGGACTGTCA	RVF_M_C1	YCTTACCATRGCAGGTGATGTTGTTCAAGC
			RVF_M_C2	ATTTGAGCCTGARATGCCCTCTGC
RVF_M2	GGAGCATCRTCTAGCCGTTTC	GCATACCCTTTGCCTGGG	RVF_M_C1	YCTTACCATRGCAGGTGATGTTGTTCAAGC
			RVF_M_C2	ATTTGAGCCTGARATGCCCTCTGC
RVF_L1	TGCGTGAGTTTCCCATGA	CTGCCCTGAGATCTGTTCTCAC	RVF_L_C1	CCCCTAAARGTGGTGAACTCAACGATGTT
			RVF_L_C2	ATGCACCYCTTTCATCTCCCCTA
			RVF_L_C3	TCYCCTCTCACATCTARTCCCTGAAGA
RVF_L2	CTRCCTCCCTGGYTGTCC	GCATCATCGTGCATCCTCTCAA	RVF_L_C1	CCCCTAAARGTGGTGAACTCAACGATGTT
			RVF_L_C2	ATGCACCYCTTTCATCTCCCCTA
			RVF_L_C3	TCYCCTCTCACATCTARTCCCTGAAGA

To demonstrate that it is not necessary to curate the sequences into separate target files for each segment, we also designed a multiplex set of primers and probes to detect all sequences from all segments of RVFV in a single reaction (Table 3). We used run_PriMux_triplet with the same primer parameter settings as above. L, M, and S segments were all input as a single target file with 198 sequences. These non-homologous sequences do not align, so a standard MSA approach to primer design with the full 198 sequences was not attempted. We predicted the targets detected by each primer/probe combination using TaqSim (http://staff.vbi.vt.edu/dyermd/publications/taqsim.html) and summarized the results in Table 4.

**Table 3:** Primer and probe (IO) sequences for RVFV run with all segments in a single input file.

Forward (F) or Reverse(R) Primer and Probe (IO)	
1|F	ATTTTCTTGGCATCCTTCTCCC
2|R	AGCAGTGGACCGCAATGA
3|F	GCCGTTTCACAAACTGGG
4|R	ATTGCATACCCTTTGCYTGGG
5|F	AATCATGGAGGGCTTTGTCT
6|R	AACATGCCACCCCAGGAT
7|F	TGACATGATGCAAGGATCAGAT
8|R	TACACTCCCAGCTCCTTC
9|F	TGACAGTCCTTCCAGCCT
10|R	AGCAAGACATCAAAACTGCTAC
1|IO	GCAAACTCTCGGACCCACTGYTCAAT
2|IO	TTTGCTTTGGCACCTGTTGTHTTTGCTG
3|IO	TCCCAAARCCTCACAAGATGACCTC
4|IO	AGAAGGTTCTCACCAGATGCAAAGTGG
5|IO	ACATTGACGGGATGACTCAGGRGGATG
6|IO	CCCTRCGRGCATCAAAYCCTTGAT
7|IO	CTCACTGGACGCAGAGGGCATYTC

**Table 4:** Numbers of RVFV targets detected by each primer/probe combination.

F	IO	R	# targets detected	% of sequences detected from this segment	segment
1		2	84	100%	S
1	1	2	83	99%	S
1	6	2	65	77%	S
3		4	59	98%	M
9		10	59	98%	M
9	2	10	58	97%	M
3	7	4	53	88%	M
9	5	10	50	83%	M
5		6	52	96%	L
5	3	6	52	96%	L
7		8	50	93%	L
7	4	8	29	54%	L

F,P, and IO numbers refer to the sequences in Table 3.

Third, we designed signatures for Dengue virus detection from 2863 genomes, consisting of 1233, 885, 644, and 88 genomes of serotypes 1, 2, 3, and 4, respectively (Table 5). These sequences show very limited homology at the nucleotide sequence level (only 28% of bases show consensus in an alignment, with no conserved regions long enough for a primer). Attempts to design primers for this target set with other alignment-based software [6], [7] (and unpublished, in-house tools, C. Torres, personal communication) failed. The Dengue alignment using MUSCLE [12] was 11,853 bp, and other degenerate primer design programs typically can handle alignments only up to 1–2 kb. We predicted the targets detected by each primer/probe combination using TaqSim and summarized the results in Table 6. Parameter settings were the same as listed above for the RVFV calculations, except maximum amplicon length = 300 bp.

**Table 5:** Multiplex set of primers and probes to detect all 2863 Dengue virus genomes.

1|F	GGTTAGAGGAGACCCCTCC
2|R	TCCCAGCGTCAATATGCT
3|F	GATYTCTGATGAACAACCAACG
4|R	TCTCTTCGCCAACTGTGA
5|F	CGCCTTTCAATATGCTGAAACGC
6|R	ATCCCTGCTGTTGGTGGG
7|F	YACCAACATGGAAGCCCA
8|R	CATCCTYTTGAAGGTTCCCATTGT
9|F	TGCGGAACCAGAAACACC
10|R	GTCATTGCCATCTGTGTCACC
1|IO	GGACYAGAGGTTAKAGGAGACCCC
2|IO	CRACCGTCTTTCAATATGCYGAAACG
3|IO	CGTGAGAAACCGTGTGTCAACTGGA
4|IO	GCCCTDGTGGCGTTCCTTCGTTTCC
5|IO	GCGTCGAAAGGCTRAAAAGAATGGCA
6|IO	TYAGACAGATGGAGGGAGAAGGARTCT
7|IO	AAGGACYAGMGGTTAGAGGAGACCCC
8|IO	CATCACYRACAAAACGCAGCAAAARRG
9|IO	CYTTGTGGCGTTCCTTCGTTTCCTAACA
10|IO	AGGAGGAGCATADTTCAACATGGCACT
11|IO	GGCCCTTGTGRCGTTCCTTCGTTT
12|IO	CCCTWGTRGCGTTCCTYCGTTTCC
13|IO	YRMTGGAAGGACTAGMGGTTAGAGGAGAC
14|IO	GCCYTKGTGGCDTTCCTTCGTTTCCTAAC
15|IO	GGCTCGACCGTCTTTCAATATGCYGA

**Table 6:** Primer and probe combinations from Table 5, and number and serotypes of Dengue virus genomes detected by each primer/probe combination.

FP	IO	RP	# targets detected	Serotypes detected	% of targets detected in indicated serotype(s)
1		2	2356	1–4	82%
1	1	2	2334	1–4	82%
1	7	2	2329	1–4	81%
1	13	2	779	2	88%
1	8	2	80	4	91%
3		4	1842	1,3	98%
3	2	4	1208	1	98%
3	15	4	922	1	75%
3	3	4	627	3	97%
5		6	840	2	95%
5	14	6	778	2	88%
5	4	6	554	2	63%
5	12	6	480	2	54%
5	11	6	469	2	53%
5	9	6	447	2	51%
7		8	1035	1,2	49%
7	5	8	791	1	64%
7	6	8	42	2	5%
9		10	611	3	95%
9	10	10	454	3	70%
3		6	61	2	7%
3	12	6	58	2	7%
3	14	6	58	2	7%
3	11	6	57	2	6%
3	4	6	57	2	6%
3	9	6	55	2	6%

### Rift Valley Fever virus signature testing

#### Virus Culture

All culture was conducted at the Biosafety Level 4 (BSL-4) facilities at the University of Texas Medical Branch and titered viral RNA kindly provided by Dr. Alexander Freiberg's group. The RVFV signatures designed by PriMux as well as those designed using more traditional bioinformatics tools (see below) were tested on total nucleic acid extracts generated from RVFV grown on Vero C1008 [Vero 76, clone E6; Vero E6] cells (ATCC # CRL-1586) using standard viral culture methods [13] and virus generation determined by cytopathic effect (CPE). RVFV titers were determined by standard plaque assays on Vero E6 or Vero CCL-81 (ATCC # CCL-81) cells [13], [14], [15].

Briefly, cells were cultured in T25 flasks under 10% media at 37C with 5% CO2 until 90% confluent. At total of 1 ml of viral inoculum (1∶200 dilution of virus stock in 2% growth media) or negative control (1 ml of 2% growth media with no virus) was used as inoculums and incubated for 1 hour at 37C, 5%CO2. Volume of media was brought to 15 ml total with 2% growth media and incubated for 2–5 days until 70% CPE was observed by inverted microscope examination. Viral titers were determined by plaque assay using standard procedures [14]. Cultured viral supernatant was clarified by low speed centrifugation and diluted 1∶3 with Trizol LS reagent (Life Technologies, Carlsbad, CA). Total RNA was extracted from viral samples and tested by attempted culture for 14 days to verify the absence of viable virus prior to viral samples being removed from BSL-4 and shipped to LLNL under Trizol-LS.

#### PCR Primers and Probes

Oligonucleotide primers and probes were purchased as lyophilized pellets from Biosearch Technologies, Inc. (Novato, CA). Purification of each lot was assessed by mass spectrometry. TaqMan (TradeMark) probes were designed with a 5′ Fam and a 3′ Black Hole Quencher molecule. Upon receipt, oligos were reconstituted in sterile 1× Tris-EDTA (TE) Buffer (10 mM Tris-Cl, 1 mM EDTA, pH 8.0, Teknova, Hollister, CA) to a concentration of 100 mM. Working stocks were made by diluting primers and probes to a concentration of 10 mM with TE Buffer. The primer and probe working stocks were stored at 4°C. Unused 100 mM primers and probes were stored at −80°C.

#### RT-PCR

All assay reactions were carried out on 96 well FAST PCR plates (Applied Biosystems, Foster City, CA) in a total volume of 25 µl (20 µl master mix plus 5 µl sample) optimized for Real-Time RT-PCR. A volume of 20 µl Real-Time RT-PCR master mix (AgPath-ID™ One-Step RT-PCR Kit, Cat#4387391, Applied Biosystems, Foster City, CA [Life Technologies, Carlsbad, CA]) contains: 12.5 µl 2× RT-PCR Buffer Mix, 1 µl 25× RT-PCR Enzyme Mix, 1.0 µl primer mix (0.4 µM each forward and reverse primer final concentration), 0.5 µl TaqMan probe (0.2 µM final concentration), 5.0 µl PCR water (Teknova Inc), and varying amounts of template RNA. Each plate contained 3 negative (no template controls) and 3 positive controls containing 1000 copies of Alien-armored RNA for each signature. Alien armored RNA is heat lysed prior to addition to the mastermix by diluting the Alien-armored RNA to 200 copies/µl in water and placing in a heat block for 3 minutes at 75°C. The Alien-armored RNA (XenoRNA-01, Ambion, Austin, TX) is a proprietary 1070 nucleotide RNA transcript consisting of unique nucleotide sequences that possess no significant homology to the current annotated sequences in commonly used sequence databases.

Reactions were carried out on ABI 7500 thermal cyclers (Applied Biosystems, Foster City, CA) under the following quantitative Reverse Transcriptase Fast thermal cycling conditions: 45°C for 10 minutes for cDNA synthesis, followed by 95°C for 10 minutes for inactivation of the reverse transcriptase, activation of 25× RT-PCR Enzyme Mix, and denaturation of the RNA/cDNA hybrid; followed by amplification at 40 cycles of 97°C for 2 seconds and 60°C for 30 seconds. Data acquisition was performed at the annealing step, and limit of detection results are reported in Table 7.

**Table 7:** Per Signature Limit of Detection for Rift Valley Fever Strains Tested: All tests were conducted on total nucleic acid extracts from clarified viral cultures containing the listed strains.

		Plaque-forming Units (pfu)*						Picograms total RNA**
	SignatureName	SA75	Ken 58(B691)	MauritaniaOS-1	ZH548	ZH501	SA51	MP-12
Degenerate	SC1	0.10	1.0	0.001	100.0	1.0	0.01	0.10
	SC2	0.10	1.0	0.001	100.0	1.0	0.10	0.10
	M2C2	0.10	1.0	0.001	10.0	0.10	0.01	0.10
	L1C1	0.001	0.10	0.001	100.0	1.0	0.001	0.10
	L1C2	0.01	0.10	0.01	1000.0	1.0	0.001	0.10
	L2C3	1.0	1.0	0.01	1000.0	1.0	1.0	1.0
Non-degenerate	1756318	0.10	1.0	0.10	10.0	0.10	1000.0	0.10
	1756321	1.0	100.0	1.0	100.0	1.0	1000.0	1.0
	1756325	0.10	1.0	0.01	10.0	0.10	1000.0	0.10

QRT-PCR tests were performed in triplicate on the ABI7500FAST platform as described in materials and methods. An 8-log dilution series of templates ranging from 200 pfu/ul to 2×10^−4^ pfu/ul (or 20 pg/ul to 2×10^−6^ pg/ul for MP-12) was made for each template. Five microliters of each dilution was spiked into qRT-PCR plates in triplicate.

* A plaque-forming unit (PFU) is a measure of the number of particles capable of forming plaques per unit volume, such as virus particles. It is a functional measurement rather than a measurement of the absolute quantity of particles: viral particles that are defective or which fail to infect their target cell will not produce a plaque and thus will not be counted.

** For RVF MP-12, no titer information is provided as multiple attempts to titer this species failed due to lack of consistent CPE. Thus, sensitivity of this assay is reported in pg of total nucleic acid extract from viral culture. No other RNA (such as poly-A RNA used as carrier in extractions) was spiked into samples. For the degenerate signatures, only the primer/probe combinations that were able to detect all strains of RVFV tested are listed.

Primer/Probe Sequences: Table 2 lists oligonucleotides with incorporated degeneracies designed using the PriMux system. Sequences of non-degenerate oligonucleotides designed using our traditional signature prediction software (described in [11]) (Signatures 1756318, 1756321, and 1756325 in Table 7) are not listed as they are proprietary and we were unable to obtain permission to publish signature sequences. All signatures (degenerate and non-degenerate) were designed to have optimal annealing temperatures of 60°C to 65°C enabling testing of all signatures using a single thermal cycling protocol as listed above.

#### Nucleic Acids Extraction

All template RNAs were extracted with three times the volume of Trizol (Invitrogen, Carlsbad, CA), mixed, and incubated 15 min at 25°C. One-fifth of the total volume of chloroform was added, mixed, incubated 15 min at 25°C and centrifuged at 3000× g 15 min at 4°C. To the aqueous layer, 70% volume of 100% isopropyl alcohol was added. The sample was mixed, incubated 10 min at 25° C, and then centrifuged at max g for 10 min at 4°C. The pellet was washed with 70% ethanol and then centrifuged at max g for 10 min at 4°C, air dried briefly at room temperature, dissolved in RNase-free, DEPC treated water (Ambion, Austin, TX) and stored at −80°C until needed.

The concentration of extracted RNA was determined for the samples using a ND-1000 Spectrophotometer (NanoDrop Technologies, Wilmington, DE). Final titer amounts were determined using the titer information provided by UTMB for the total nucleic acid extracts from virus culture stocks provided.

#### Specificity Testing on Target, Near Neighbor and Background Nucleic Acids

A collection of purified RNA samples was used for signature testing and down-selection. Part of this collection was generated and generously provided by the Freiburg laboratory at the Center for Biodefense and Emerging Infectious Diseases at the University of Texas Medical Branch (UTMB, Galveston, TX) and included RNA extracts from a total of six titered targets (strains SA75, Ken58 (B691), Mauritania OS-1, ZH548, ZH501, SA51) and one un-titered target (strain MP-12), and 16 genetic near neighbors of Rift Valley Fever (RVF) virus strains (from 3 genera and 13 species from the Bunyaviridae family). Near-Neighbor strains are defined as genetically closely-related organism that are phylogenetically similar (related) to and yet distinct from our target organism. Each signature was screened in triplicate against the target and near neighbor RNA extracts. All initial target reactions were performed using an amount of total RNA extract that was equivalent to 1000 pfu total RNA from titered viral samples. For the untitered MP-12 (vaccine) strain, 100 pg of total RNA extract was used. All near neighbor reactions were performed using 1 ng total RNA extracts from viral culture samples generated as RVFV. Each signature was additionally screened against a collection of “background” nucleic acids (nucleic acid extracts from a variety of mammalian, avian, and arthropod cell lines). This panel is designed to challenge the specificity of each signature, ensuring that the signatures react only with target nucleic acids and not the nucleic acids from various potential viral hosts. RNA extracts were quantitated on an ND-1000 Spectrophotometer (Nanodrop, Wilmington, VT). Each signature was screened in triplicate against background cell lines. Background reactions were performed using 200 pg total RNA.

#### Titrations to Determine Limits of Detection

All signatures that were down-selected were tested using eight-fold serial dilutions of extract RNA from six titered and one untitered Rift Valley Fever strains in the previously described Real-Time rt- PCR format. The total amount of titered and untitered template added to each reaction ranged from 1000-0.0001 pfu and 100-0.00001 pg total RNA. The reactions were performed in triplicate. The limit of detection (LOD) for each signature was defined as the minimum amount of target template required to generate a Ct value equal to or smaller than 35.

The clinical relevance of LOD is virus- and application-specific. As molecular assays detect only the target nucleic acids and not infectious virions, they cannot differentiate between infectious and non-infectious targets detected, and it is important to know the ratio of viral genomes to infectious particles they represent. As such numbers have not been readily available, most molecular assay development focuses on generating the most sensitive assays possible. New research does shed some light on the ratio of infectious particles to number of genomes present [16]. Weidmann and colleagues found a vast range in the ratio of viral genomes versus infectious particles with Flaviviruses and Arenaviruses being the most efficient while Phleboviruses such as RVFV generated a large surplus of RNA genomes per infectious particle produced. Thus, given those results, while assay sensitivity may not be critical to efficient and effective diagnosis of RVFV infection, it is of great importance in detection of Yellow fever, Dengue and Lassa for example. A further complicating factor is that controlled studies in animal models of infection that would conclusively determine infectious dose and thus diagnostic utility of any molecular assay are difficult to conduct with highly dangerous pathogens and pose obvious ethical issues if attempted with humans, thus the paucity of information demonstrating clinical relevance at least for the most dangerous viruses.

## Results

Taqman signatures designed with PriMux for each RVFV segment included 1 or 2 primer pairs and 2–3 internal oligo probes per segment. Sequences from one of the several possible solutions for each segment are listed in Table 2, and these were tested in the lab. We tested signatures from the solution set predicted using the “max” algorithm for segment S (2 primers and 2 probes), the “min” algorithm for segment M (4 primers and 2 probes), and the “combo” algorithm for segment L (4 primers and 3 probes). These were not necessarily the solutions with the fewest primers or probes: for example, the max and combo algorithm produced solutions requiring only 2 primers to detect all strains of the M segment, for example, with primer sequences F = GCCGTTTCACAAACTGGG and R = GAATGGCTCATCAACAATTGCA and probes = GTYAGCCTCTCACTGGAYGCAGA and GACGCAGAGGGCATYTCAGGCTCAAA.

Running all RVFV segments together as a single target file predicted a solution set with 10 primers (5 pairs) and 7 probes (Table 3). The solutions from running the segments together are not identical to those from running them separately due to both a random selection of equivalent k-mers (in terms of conservation and passing the filters), because of the effects of amplicon length and primer dimer avoidance, and because the greedy algorithm does not explore every possible solution. The number of targets detected by each primer/probe combination and the segments detected are shown in Table 4. All S segment sequences are amplified by the (1|F, 2|R) primer pair, and all but one of the S sequences is detected by the 1|IO probe. To detect the last strain SPU45ZAMB85 (gi|168013438), PriMux selected an additional probe 6|IO, which also detects a number of other sequences. Similarly for segments M and L, there is a primer pair and probe for each that detects the majority of targets of that segment, but a couple of extra primers and probes are needed to detect some outlier sequences that are not picked up by the majority probes (e.g. gi|87622799 is only detected by probe 7, and gi|87622819 is only detected by probe 5). If a user is willing to miss a few of the outliers, a single triplet per segment could detect 96–99% of the sequences.

For Dengue virus, we found solutions with as few as 10 primers (5 pairs) and 15 probes (Table 5). In previous work we had found SCPrimer [6] and Hyden [7] scaled the best for degenerate primer design of larger target sets, although neither predict internal oligo probes. In this case, Hyden produced error messages that the sequences were too long, while the web site for SCPrimer was not working, and we could not ascertain if the site is still maintained. In previously saved calculations from the SCPrimer website against the smaller, less diverse set of 870 Dengue serotype 2 sequences, the best solution (fewest primers to detect all sequences) we found had 9 primers. Our own unpublished, in house software for degenerate primer and probe prediction from an MSA also crashed on this very large and diverse target set before signatures could be found (C. Torres, personal communication). In Table 6 we report the numbers of targets and the serotypes detected by each primer and probe combination. At the bottom of the table, it shows that although primer 3 was designed to pair with primer 4, and primer 5 with 6, in a multiplex, primers 3 and 6 may pair with one another to amplify a small number of targets. As in the RVFV example, the best triplet detects the vast majority of sequences, or 82% of all Dengue genomes. Adding primers and probes to the mix ensure detection of smaller outlying groups of sequences. For each serotype, there is a particular triplet that detects 88–98% of the targets. So in this case, the PriMux solution to detect all sequenced Dengue genomes contains a combination of serotype specific and multi-serotype primers and probes.

### Lab testing

To demonstrate that signatures designed with PriMux performed as well as those designed using a standard multiple sequence alignment approach as in [11], TaqMan™ signatures PriMuxwere tested on RVFV target and near-neighbor nucleic acid extracts. Note that PriMux does not consider signature uniqueness relative to a database of non-target sequences, so we always computationally check signatures designed with PriMux for target specificity using BLAST comparisons against Genbank as implemented in TaqSim (http://staff.vbi.vt.edu/dyermd/publications/taqsim.html).

Table 7 lists the primer “S, M, or L” and the probe “C” combination from each set of signatures listed in Table 2 that were able to detect all target strains. No primer/probe combinations (those listed in addition to those not able to detect all targets) tested resulted in any cross-reactivity with any non-target nucleic acid. Results are reported for Limit of Detection studies conducted in triplicate reactions for each signature/target combination. Cycle threshold cutoffs were 35 (ie: any Ct greater than 35 was defined as a detection failure. Thus, for each RVFV strain tested, the LOD reported in Table 7 is the template concentration at which all three replicates of the RT-PCR reaction generated a Ct value equal to or below 35.

The M1C1 primer/probe failed to detect one RVFV strain, ZH-548. This strain has been sequenced, and is predicted to be amplified by that signature, as the primers and probe match the genome. We do not know why it failed to detect this strain. It could be that the sample we had in the lab was a mutated variant of the genome sequence in Genbank for positions in that triplet, or a titer issue. The other M segment signature does detect this strain.

Solution sets are intended to be used in combination: a single primer pair and probe in a solution is not expected to detect all targets, but in combination all the primers and probes in a PriMux solution should detect all targets. Recall that the M segment solution that we tested was generated with the “min” algorithm, which required 4 primers to detect all strains, rather than the set of just 2 primers conserved across all strains from the “max” algorithm.

The fact that each primer/probe triplet tested except M1C1 detected all targets in our sample collection argues that our collection does not represent the total sequence diversity of those strains that have been sequenced. Obtaining a wide array of samples is a challenge. This is an issue for everyone who develops diagnostic signatures, and every sample collection most likely has strains missing compared to worldwide diversity for a given species. We rely on *both* the available sequence data and any lab isolates we can obtain for screening diagnostic signatures, although this still does not guarantee detection of novel isolates.

The results in Table 5 demonstrate that the degenerate signatures designed with PriMux performed at least as well as the non-degenerate signatures that we designed previously using more standard MSA and consensus methods for detection of SA-75, KEN58, ZH501, and MP-12 strains of RVFV. The non-degenerate signatures were able to detect the ZH548 strain 1–2 logs better than two of the degenerate signatures (L1C2 and L1C3), but L1C2 and L1C2 detected two other strains (Mauritania OS-1 and SA51) by up to 6 logs better than the non-degenerate signatures, and the performance of each of the other 4 degenerate signatures was essentially equivalent to that of the non-degenerate signatures.

## Discussion

We describe PriMux, a k-mer based approach to designing primers, probes, and Taqman® triplets for detecting diverse sets of target sequences, even those that contain non-homologous sequences. No multiple sequence alignment is required. Instead, a greedy algorithm based on k-mer analysis with suffix arrays identifies conserved, degenerate k-mers that meet primer specifications (Tm, etc.) and which can be combined in multiplex to amplify at least one fragment from each of the target sequences. k-mer based codes are also described to predict conserved or discriminating sets of probes for a set of input sequences. These input sequences can be the amplicons produced by PriMux-designed primers, resulting in primer+probe triplets for use in assay formats such as TaqMan® or Luminex®. This is the only software to design multiplex triplets to detect all members of a large, diverse, and potentially non-homologous (and unalignable) target set. It allows the user to specify a number of primer characteristics, including the number of degenerate bases allowed.

We tested PriMux signatures designed for RVFV, a Category A threat list virus that results in severe morbidity and mortality in humans and livestock [17]. Labaratory tests comparing the performance of degenerate primers and probes designed using the PriMux software to signatures designed previously utilizing traditional consensus methods demonstrated that the degenerate signatures designed by PriMux performed as well as or better than non-degenerate signatures (by up to 6 logs) designed by more traditional software approaches. PriMux also successfully designed a small set of multiplex triplet signatures to detect all members of a non-homologous target set that included all L, M, and S segments of RVFV analyzed simultaneously, as well as an exceptionally large, diverse target set containing all sequenced genomes of all serotypes of Dengue virus, with nearly 3000 sequences approximately 11 Kb in length. Other applications for which we have used PriMux are to design sequencing primers that tile in overlapping segments across a set of target genomes, signatures for a toxin or antibiotic resistance gene family, and microarray probes.

The PriMux software is available as open source from http://sourceforge.net/projects/PriMux.
